# Tea Party Health Narratives and Belief Polarization: the Journey to Killing Grandma

**DOI:** 10.3934/publichealth.2017.6.557

**Published:** 2017-11-27

**Authors:** Kristin Haltinner, Dilshani Sarathchandra

**Affiliations:** Department of Sociology and Anthropology, University of Idaho, Moscow, ID, USA

**Keywords:** political polarization, right-wing populism, Affordable Care Act, Obamacare

## Abstract

In the past decade the U.S. public has expressed varying degrees of skepticism about certain factual claims, and of “expertise” more broadly. Ideological and partisan belief polarization seems to have elevated public anxiety about topics ranging from climate change and vaccines to immigration and healthcare policy. Furthermore, polarized narratives about scientific, medical, and political topics have encouraged “directionally motivated cognition”, leading to a decline in institutional trust among some fractions of the U.S. political spectrum. Our case study of the *Tea Party Patriots* (TPP) (i.e. a political organization that promotes the Tea Party goals) uses data from 45 interviews, 80 hours of participant observation, and content analysis of movement literature, to examine the nature and nuance of health narratives employed by the Tea Party. Specifically, we explain a central narrative in TPP organizing that features “a villainous Left covertly seeking to harm U.S. citizens” as the root of three key TPP health care narratives: (1) Democratic health initiatives enslaving youth; (2) the political left profiting from covertly making Americans dependent on state's health care programs; and (3) the left clandestinely seeking to violate the constitution as represented by their efforts to “kill grandma”. These narratives reflect the increased polarization of attitudes towards healthcare, as well as a broader distrust of the political left who, activists believe, are advancing a political agenda of social control. Ultimately, we argue that culturally driven healthcare narratives of the Tea Party have had a significant impact on right-wing public opinion and Republican politics regarding U.S. healthcare policy. Many Tea Party concerns are reflected in the Republican policy positions, including those related to the Affordable Care Act of 2010.

## 1. Introduction

In a widely-viewed 2009 appearance, CNBC news reporter Rick Santelli chose to criticize then President Barack Obama, arguing, “…President New Administration, why don't you put up a website to have people vote on the Internet as a referendum to see if we really want to subsidize the losers' mortgages, or would we like to at least buy cars and buy houses in foreclosure and give them to people that might have a chance to actually prosper down the road, and reward people that could carry the water instead of drink the water…” [1].

Right-wing social movement scholars have often pointed to this moment as the beginning of the U.S. Tea Party movement. Santelli's message resonated with several right-wing organizations (e.g., *ResistNet*, *FreedomWorks*, *Our Country Deserves Better PAC*) that formed the basis of the U.S. Tea party, assisted by large financial contributions from libertarian millionaires such as the Koch brothers [2],[3]. Driven by a predominantly libertarian ideology, the Tea party's main goals include promoting fiscal responsibility, limiting government control, and bolstering free market capitalism.

Since it's origin in 2009, the Tea Party movement has not only become an integral part of the U.S. mainstream politics but also a significant “cultural force” on the nation [4]. The Tea Party has exerted its influence to shape the Republican party platform and its politics [5],[6], tempting some scholars to point to the Tea Party as the mainstream right [7]–[9]. Evidence of this assertion includes the influence of the Tea Party on Mitt Romney's 2012 presidential campaign [10], the success of Governor Scott Walker of Wisconsin in curbing the strength of labor unions [11], and cultural support for these initiatives.

During the 2016 presidential campaign cycle, the Tea Party threw its support behind Donald Trump, in addition to paving a political opportunity for his rise through shifting cultural narratives. This discursive shift includes (1) abandoning previously held values such as political correctness and intellectualism; (2) rejecting political insiders and traditional politicians; and (3) supporting policy initiatives that mirrored those rejections [12].

Political ideology is both itself a cultural artifact and manufactured from one's deep culture. Political ideological systems consist of culturally shared beliefs and values [13] and people use their culturally rooted perspectives to develop political positions [14]. Moreover, one develops their political beliefs with and through socialization and acculturation [15]. Unlike other cultural artifacts, adherents generally seek opportunities to share political narratives. Given these factors, the expression of political narratives regarding health care provides a rich public lens from which to view and analyze the cultural scripts of Tea Party members.

Given this backdrop, we argue that the *Tea Party Patriots* (TPP)—a major political organization that promotes the Tea Party goals—provides an avenue where scholars can examine how ideological and partisan divisions, as reflections of deep culture, drive belief polarization through political narratives, which may carry implications for policy making in U.S. politics. Supported by extant literature on belief polarization, we demonstrate how the TPP consists of a specific population that sustains and perpetuates healthcare narratives driven by a predominantly libertarian ideology. Specifically, we examine a central narrative in TPP organizing that features a villainous Left covertly seeking to harm U.S. citizens, which we identify as the root of three health care narratives in the TPP: Democratic health initiatives enslaving youth, the political left profiting from covertly making Americans dependent on state's health care programs, and the left clandestinely seeking to violate the constitution and eliminate vulnerable Americans as represented by their efforts to “kill grandma”. Ultimately, we argue that the culturally driven healthcare narratives of the Tea Party have impacted Republican politics regarding U.S. healthcare policy.

### 1.1. Belief Polarization in the American Public

Political beliefs are deeply rooted in one's cultural environment and are reflective of their cultural ideologies. People develop a “cultural worldview” as a result of both internal processes and the belief structures—including political ideologies—of the culture in which they reside [16]. Political positions are often outgrowths of one's cultural worldview [17]. The visible manifestations of political beliefs and political ideologies as cultural narratives provide a keen opportunity for analysis.

Beliefs are polarized when presentation of the same information elicits divergent attitudes between people [18]. In recent years, polarization of attitudes towards science, health, and policy topics has been on the rise among the American public [19],[20] as seen by debates surrounding abortion rights [21], anthropogenic climate change [22], and vaccine safety and efficacy [23]. These trends have prompted some scholars to point to partisanship and ideology as the main drivers of belief polarization.

Political scientists employ the *party sorting theory* to explain belief polarization in politics. Accordingly, political party elites and activists drive ideological divisions between Democratic and Republican constituents by gradually transferring the elite political polarization to the general public, to the point where, the two parties become “increasingly divided on all the major policy dimensions in American politics” [24]. Party sorting is a “top-down process wherein the more visible and active members of a party, especially its elected officials and party activists, sort first and provide cues to voters that party positions are evolving” [25].

In the realm of science and health, belief polarization results in increasing politicization of scientific topics and distrust of scientists, among both the political right and the left, though the effects seem to be greater on the right [26]. In examining the effects of politicization of science, Gauchat employs the *politicization thesis* to demonstrate that, in fact, politically conservative subjects experience comparatively larger, long-term group-specific declines in trust of science/scientists compared to those who are politically liberal. As scientific authority is driven by the perceived political neutrality and objectivity of scientists, any deterioration of trust in scientific authority may trickle down to the level of science-based policy, including policy on public health.

Healthcare reform debates in the U.S. provide evidence for how scientific and factual claims become polarized when adapted as policy and/or applied into the public sphere. For example, two recent Democratic presidents, Bill Clinton (1993–1994) and Barack Obama (2009–2010), both faced intense divisions in ideological and partisan viewpoints, fueled by widespread misperceptions (e.g., Clinton doctor choice, Obama death panels), during their respective attempts to reform the U.S. healthcare system [27]. The current Republican administration, lead by President Donald Trump, seems to be facing an equally polarized political climate as it attempts to reform healthcare.

### 1.2. Causes and Effects of Ideological and Partisan Belief Polarization

Belief polarization is rooted in a psychological phenomenon known as “directionally motivated reasoning,” where individuals process information in ways that allow them to reach desired conclusions [27]. This type of cognitive reasoning “leads people to seek out information that reinforces their preferences (i.e., confirmation bias), counter-argue information that contradicts their preferences (i.e., disconfirmation bias), and view pro-attitudinal information as more convincing than counter-attitudinal information” [28]. In politics, directional cognition is often moderated by individual and contextual level factors. Examples include one's cultural worldviews, political knowledge, media choice, issue salience, elite partisan polarization, and, in-group/out-group effects [29].

Notably for our study, the most common sources of directionally motivated reasoning are partisanship and ideology, which are both related to one's cultural worldviews [29]. When assessing affectively-charged hot-button issues such as healthcare reform, the motivations driving the reasoning are more likely to reinforce existing political party loyalties and ideologies, and affirm preexisting beliefs about “how society should operate” [30], rather than compelling constituents to seek out fact-based information [31].

Furthermore, affect-laden and controversial policy issues inhabit fertile grounds for the spread of “political misperceptions” (i.e. “demonstrably false claims *and* unsubstantiated beliefs about the world that are contradicted by the best available evidence and expert opinion”) [27]. When it comes to political misperceptions, Nyhan shows that partisans do not simply differ in their views about political issues, but they also differ in “factual beliefs” about the status of the world, and those misperceptions often skew in partisan directions (For example, polling data shows that while a higher percentage of Republicans believe that Obama was not born in the U.S., a higher percentage of Democrats believe that the Bush administration officials were complicit in the 9/11 attacks).^1^

Political misperceptions are deeply troubling, because they distort public opinion about important issues related to health, science, and politics. Making matters worse, affect-laden political misperceptions (such as the death panels myth) are often resistant to change, especially if the factual information challenges a person's existing worldview [27],[30]. When political misperceptions are highly salient, providing corrective information fails to change the inaccurate original beliefs. In some cases, this may even backfire and make the said misperceptions worse (“backfire effect”) [29],[31],[32]. Furthermore, recent research suggests that the backfire effect is more pervasive when it involves information that challenges one's preexisting cultural worldviews [30].

## 2. Materials and Methods

Data for this case study includes 45 in-depth interviews with members of the TPP collected between 2010 and 2012; participant observation at two Tea Party chapter meetings, conducted between 2010 and 2012; and content analysis of official content and editorials on the Tea Party Patriots website (www.teapartypatriots.com) drawn from 2013 to 2016. The TPP was chosen due to its size (self-estimating over 1,000 groups), self-proclamation as the “umbrella organization” for the Tea Party movement [4], and accessibility.

Interviews ranged from 45 to 90 minutes in length. Data is drawn from a convenience sample of state directors and rank-and-file members from thirteen states nationwide: California (n = 1), Illinois (n = 8), Iowa (n = 1), Massachusetts (n = 1), New Hampshire (n = 1), New York (n = 2), North Carolina (n = 2), Ohio (n = 1), Tennessee (n = 1), Texas (n = 1), Virginia (n = 4), and Washington (n = 1), but primarily Minnesota (n = 21).

Forty percent of interviewees were recruited at chapter meetings. Contacting activists from different Facebook TPP groups led to an additional 30 percent, most of whom were interviewed via Skype. The remaining third were contacted through email addresses obtained from the national TPP website. These interviews were conducted in person and via Skype. Names have been changed to protect the identity of participants.

Participant observation was conducted at the regional meetings of two chapters in Minnesota as well as locally-run workshops and events. One chapter routinely hosted 60 participants at weekly meetings in a community center while the other hosted 300 attendees for monthly meetings at a bar. These chapters were selected to capture perspectives from rural, suburban, and urban membership.

**Table 1.** Demographic Information of the Interview Sample.

*Total sample (N=45)*	
*Gender: % female*	31.1
*Age: median*	55
*Race: % White*	96.0
*Income: median*	40,000

The ages of participants roughly form a bell curve with a median age of approximately 55. This mirrors Lundskow's finding that activists tend to be either retired or younger business owners [33]. In the sample, members work in a variety of careers including as lawyers, realtors, administrative assistants, business executives, and civil servants. Four are unemployed. The median income for an individual in the sample is $40,000, near the individual median income for the nation as a whole at $42,693 [34]. Previous work suggests that TPP activists are primarily older, white, and middle class and, thus, weathered the recession better than many Americans [4],[35],[36]. However, TPP members tend to be in a financial situation whereby they lack significant wealth yet are unable to receive social welfare services [4]. As a result, they feel attacked from both people below them in the social hierarchy benefitting from such programs, and perceived elitists who make the policy decisions that fund social welfare programs [37].

Most participants are white (96 percent). The exceptions include Wayne, who is black, and Aaron, whose father is Japanese and mother is white. The sample is reflective of broader TPP demographics in the state of Minnesota and nationwide, where it is estimated that 91.4 percent of TPP members are white [4],[35],[36].

IRB approval was received from the University of Minnesota. Consent was given orally, as approved by the IRB, in order to best protect the identity of participants. Interviews were semi-structured: they followed an interview guide, but were conversational. Interviews began by asking participants to tell the story of how they came to join the TPP, probing into the factors that contributed to their membership. To examine ideas about race, gender, and class, participants were asked what they saw as the biggest problems facing the United States today. The conversation was then directed towards issues of Medicare, Medicaid, the ACA, and other relevant topics. All interviews were recorded and transcribed verbatim.

Content analysis was conducted on documents found on the Tea Party Patriots website. All of the official documents found under the “health care” tab were coded and analyzed. Additionally, the website provides a search feature. Researchers searched for and coded data found using the terms: health care, healthcare, Obamacare, Affordable Care Act, science, and scientific. When using the term health care the search provided 512 articles. We included every tenth article in our analysis. The other terms resulted in smaller numbers of articles; each was included in the analysis. In total we included 72 articles in our analysis. Emerging from this analysis are the three narratives outlined below, what we call, enslaving youth, profiting from social parasitism, and killing grandma.

Data were coded for patterns and negative cases using standard inductive analysis [38] through Atlas TI. To analyze these patterns, the interpretive paradigms of ethnomethodology and poststructural discourse analysis were used to analyze both a local practice and broader social context [39]–[45].

Limits to this methodology include the use of state directors as primary interview brokers. Although this may have filtered some interviewees, in some states, interview participants self-selected into the study after receiving an email sent to a statewide listserv, reducing concern of censorship. Self-selection could lead to people with stronger views dominating the sample. Secondly, the general distrust of academics among right-wing groups presumably shaped the information members provided [46]. However, the range of responses suggests that participants generally felt comfortable and other scholars [47]–[49] have similarly found that right-wing activists are happy to share their views with a wider audience. An additional shortcoming comes from the use of Skype interviews, rather than meeting activists in person, as this may limit rapport and disclosure. Finally, given that the sample was only members of the TPP we are unable to examine the degree to which these health narratives mirror or extend those employed by other conservative and/or mainstream Americans.

## 3. Results and Discussion

Tea Party health narratives emerge from and reflect a broader identity narrative produced by the movement and the political-right more generally. As a populist movement, the Tea Party's central narrative reflects the broader populist “producerist” narrative [37]. Producerism is the perception that true Americans work hard and produce despite having to fend off the threats of “parasites at the top and bottom of society” [37]. The Tea Party movement engages with these historical ideas of right-wing populism and creates a tertiary identity narrative: the antagonist (an amorphous, shifting villain embodied by “the Left”), the victim (the American citizen), and the hero (the TPP) [50]. This narrative, embraced and manufactured by the TPP, has a common place in American society and mirrors the stories of superhero movies, comic books, and Disney films: an evil force seeks power and brainwashes or enslaves the innocent population whom are saved by a central hero.

The TPP narrative is also influenced by the libertarian beliefs central to the movement's organizing. Libertarianism exists on both the left and right wing of the political spectrum, but in its conservative form generally forefronts a search for limited federal intervention regarding individual rights and the operation of the economy [51],[52]. Libertarians believe that a free market economy and political system will inevitably lead to rights and freedoms for individuals [51] and that government welfare programs will limit personal rights and freedoms by either bolstering the weak or making people dependent on state services [53].

The Tea Party, like other social movements, is host to its own culture [54],[55] and reflects an existing fissure within the broader culture of American society while concurrently trying to change the said national culture [56]. In particular, Tea Party culture merges libertarian beliefs with right-wing populist narratives. Activists then use their resultant overarching narrative to shape American culture to embrace the same.

Rooted in this broader narrative and ideological framework, the Tea Party produces three central health narratives, reflective of the overarching tertiary identity narrative. First, the Tea Party narrates that federalized health care initiatives, put forth and promoted by democrats, unjustly restricts the liberties of all American citizens, but especially healthy people, and are thus immoral. Second, the organization claims that the Democrat-promoted programs that provide health benefits hurt American citizens by creating a slovenly working class dependent on government handouts, also an immoral practice. Third, the Tea Party claims that national health care programs are unconstitutional and view the Left as duplicitously crafting illegal policies and programs—that unjustly harm Americans—for their own gain.

### 3.1. Enslaving Youth

Tea Party activists' cultural health narratives reflect an overarching narrative about leftist evil harming citizens as viewed via libertarian beliefs regarding the relationship between liberty and state coercive power. The emergent narrative subsequently argues that the federalized health care programs touted by Democrats are immoral because, through taxation, they restrict liberties for young, healthy people.

The construction of Tea Party health narratives is predicated upon a controversial libertarian argument: that as individuals or as a collective, members of a society have no direct responsibility to aid one another. The choice to provide assistance to others is one entirely left to the individual. One Tea Party activist, who we call Wayne, provides an explanation of this absent social imperative:

The answer is politically incorrect and, for many people, very offensive. And that is: nobody [is responsible for others]. I do not have a responsibility to any other human being, aside from the one that I made. I have a responsibility to that one. But, I do not have a responsibility to any other human being unless I contract out that responsibility.

Libertarians share a morality centralized on the importance of individual liberty [53]. Moral choice, then, is deeply revered as an individual option, as Wayne highlights. While Wayne's position may be, as he suggests, controversial (in fact, a 2017 Gallup poll shows that 52% of Americans believe the government is responsible for making sure all citizens have health insurance [57]), he further follows libertarian logic and suggests that individual people should employ their own value systems to determine when and how to offer aid:

If walked out to my car and I saw some little girl... about to get hit by a car, my reaction—hopefully—would be go to save her, right? Now why is that?... I'm not responsible for her. I'm not compelled to do that by some moral necessity. The answer is that I value life. I value humanity. I value that little girl, even though I don't even know that little girl... People interpret it as collective responsibility, but that isn't what it is. It is an individual judgment... And, I don't have any business telling anybody else, nor does anybody else have any business telling me, what I should value.

Libertarians generally, and Wayne specifically, reject progressive arguments that, without state intervention, those in need will go unaided.

When we've seen less intrusive government, like, 17th century America, you didn't have orphans and widows dying in the street, it just didn't happen. Because there is always going to be somebody there who values that person. Even if it's a complete stranger...

Wayne's knowledge of historical practices regarding poverty provides a clear example of partisan and ideological polarization, as it spins historical facts through a contemporary libertarian lens. During colonial America poverty was managed at the local level (what Wayne calls “less intrusive government”). The greatest concentration of those in poverty at the time included, as Wayne highlights, “orphans and widows”. However, Wayne's interpretation that individuals, rather than governmental programs, supported the poor reflects a political interpretation rooted in cultural values. Colonial American attitudes and policies toward people living in poverty mirrored those outlined in Britain's Elizabethan Poor Laws. Under this framework the poverty of able-bodied people was punished, often severely, by local communities. Care for those in poverty due to sickness, old age, or widowhood, was managed at the local level and did involve the provision of public aid [58].

At this time, the line between public and private support was quite blurry. If families were unable to help themselves, care for the poor was largely thought to be the job of local religious organizations and wealthy philanthropists. Yet, programs were often managed publicly. Local taxes were levied to provide support for those unable to work. However as this model of funding became unsustainable in the early 1700s, state governments began providing financial support to local communities to help impoverished citizens unable to work [58].

In addition, local provision of care was typically coupled with severe consequences for those needing support. Children were often taken from impoverished homes and forced to work as “apprentices”. Poor people were often barred from relocating to new communities, whom did not want to financially support them [58].

Cultural narratives, such as Wayne's claims above, need not be factual or truthful to have great influence over those who embrace them and to sway others to whom they are exposed. Rather, cultural narratives are deeply resistant to, what Heller, calls “contravening evidence” [59]. In other words, cultural narratives are not readily shifted by evidenced-based arguments as they are often flexible enough to adapt to or powerful enough to reject alternative explanations. This is the persuasive power of Wayne's claims. He argues that people living in the 17^th^ century had the “freedom” to operate according to their own values and that, as a result of a smaller federal government, this time period reflects an idyllic part of U.S. past. Wayne's story is not permeable to an analysis of the experience of people in poverty in the 17^th^ century or evidence of the decreasing distress faced by those in poverty as federal assistance developed.

Nonetheless, the fundamental cultural story, that members of a society are not beholden to care for others, forms the basis for the Tea Party claim that the ACA, and any federal health care program, unjustly harms the young and the healthy. According to the TPP website, health premiums for young people will increase by between $500 and $783 due to “this tax” included in the ACA. Tea Party activists, given their positions on individualized morality, believe that this unfairly punishes young, healthy people in the name of an erroneously directed public good. Margot refers to the Tea Party narrative of “death panels” (discussed below) and argues that the collection of money from young people to subsidize health care is even “more important” as an issue: “You don't want to cut off grandma and grandpa but, you know what is more important? Do you want your grandchildren to be paying taxes like this?” In this narrative, Tea Party members conflate medical premiums with taxes. This is significant in that members of the political right generally seek a reduction in taxes for individuals. Thus, this conflation allows the Tea Party to use partisan politics to increase resistance for the policy.

Given the value libertarians place on individual rights and freedoms, the expectations that health care is to be subsidized by healthy people is particularly egregious to Tea Party members. Employing libertarian logic, they reject the notion that health care is a human right and, instead, create a narrative that positions the Affordable Care Act as exploitative and akin to slavery. According to Marion:

I took great exception to the notion that health care is a fundamental human right because, to my mind, you can't have a right to something which requires someone else to provide it. And what annoyed me wasn't just the fact that that claim was being made but that it was not being challenged..., [but that] there was just this sort of student response of “well, I don't want to say that it's not because then I sound like I'm not compassionate or I don't care about people” instead of looking at it from principle which is if I can lay claim to a right which requires somebody else to produce something than I am effectively advocating for slavery. In my mind it is no different than, uh, somebody who has a plantation, they've got a field and they say “I need my field to be plowed therefore I have some claim that you do it.”

Marion argues that health care cannot be a right because it relies on another entity for provision. As mentioned earlier, this entity is viewed by Tea Party activists as young, healthy people who pay into a system at a higher rate than they employ it. Marion's, and the broader Tea Party's position on this issue is in deep contrast to that of other political positions, including that of the European Union and the United Nations, both of which formally view health care as a human right [60]. As with Wayne's discussion about 17^th^ century poverty assistance, Marion's definition of slavery serves as a powerful narrative not easily influenced by more nuanced explanations of the peculiar institution.

### 3.2. Profiting from Social Parasitism

The Tea Party cultural narratives regarding healthcare for impoverished Americans not only reflect broader identity narratives in the movement but also those established under the Elizabethan Poor Laws of the 1600s [61]. Expanding on narratives of colonial America, the early 1900s brought a returned emphasis among American politicians to distinguish between the deserving and undeserving poor. They believed that welfare-like programs hurt poor people by continuing a cycle of poverty and that using taxes to fund such programs was akin to stealing from the rich [61]. Poor people, in this narrative, were constructed as lazy parasites taking advantage of hard working Americans. Possessing their own culture (the culture of poverty), scholars and politicians argued that “the poor” lack morality, such as in the case of modesty and prudishness; are apathetic or passive; lack impulse control and forward thinking; and have weak moral and psychological strength [61]. Despite a slight decline in this moral narrative during the Great Depression, the argument that poor people are somehow deficient was fully restored throughout the 60's, 70's, 80's, and 90's [61].

Tea Party activists varyingly employ the in-group/out-group differences described above, to argue that state aid causes an unhealthy dependence on the state by marginalized populations. Leonard suggests that the state has intentionally manipulated poor people generally, and black people specifically, through the welfare system. Prior to receiving state aid, Leonard argues, black women were often forced to “take back” abusive husbands but, after having access to welfare, women respond by rejecting their returning husband. As a result state aid has destroyed the black family: “he goes back to his friends and he has idle hands now, no job, no family to care about, and sons that are growing up without fathers and seeing this is their only option.” Thus, it is welfare to blame for urban poverty: “the welfare guaranteed that they'd stay in the hood forever and they wouldn't be integrated. The public housing would then go up in the same bad neighborhoods.”

Regarding the Affordable Care Act in particular, Virginia argues that the provision of health care to Americans also undermines the family: “Our families have fallen apart… Italy and Spain—an egregious amount of 30 year olds are living with their parents still. They have grown up under the welfare state. They know no other lifestyle than being supported by a government. So, they don't go out and get jobs. Their state pays for their health care… So that is where things are starting to fall apart.” With this example, we again see how Tea Party narratives about the danger of government subsidized heath care trumps factual counter arguments. While it is true that nearly 80% of Italians between the ages of 18 and 30 live with their parents (vs. 65% of Spanish and 43% of U.S. people of the same age), scientific analysis has shown that this trend is a result of cultural differences, a lack of job opportunity, and exorbitant housing costs, rather than government health initiatives [62],[63].

Tea Party activists narratives reflect a belief that Democrats seek to make poor people and other marginalized groups dependent on government aid in order to gain political support and coercive power. Leonard specifically argues that Democrats are using black people as “a pawn in a game that they are not even aware of.” Leonard suggests that Democrats “always had a sullied name with black people” and that, as a way of fixing this relationship, they decided to establish the welfare system. According to Leonard: “They thought: ‘here is a way to win them back, give them free taxpayer money.’”

Regarding health care as a specific form of social aid, the Tea Party replicates the cultural narrative of the easily duped undeserving poor. For example, Peter argues: “They [Democrats] are trying to get more people on Medicaid. In Chicago, whether [the Medicaid subscribers] are legal or illegal, they don't care… Those people [undocumented immigrants] are on Medicaid right now are destroying us… They [Democrats] live off of people that are dependent on government. So they have to have these people dependent on government.”

Official documents provided by the TPP support Peter's claim. Drawing off of a report from the Congressional Budget Office, the Tea Party argues that: “Obamacare creates a “disincentive for people to work” and “lowers economic growth.” The TPP further reports that the CBO “estimates a decline in the number of hours worked due to Obamacare equivalent to about 2.0 million full time workers in 2017.” To make their argument the Tea Party filters the CBO report ideologically, failing to mention that the CBO believes three quarters of that decrease would be made up of people who no longer will need to work to acquire health care from an employer and will thus choose to exit the workforce [64]. They also fail to mention that this decrease is only a projected loss of 0.86%, choosing instead to frame their argument in a more polarizing way.

### 3.3. Killing Grandma

One of the more well known health tropes to emerge from the Tea Party is the oft repeated fear that the Affordable Care Act would construct “death panels” which would serve as an evaluative committee, positioned to determine the value of an individual and their worthiness to live. Emerging from this claim, Tea Party activists and national politicians elected by the Tea Party developed a narrative that the ACA would lead to state sanctioned murder of the elderly; the killing of society's grandmas. Specifically, the narrative argued that the Obama Administration sought to deny medical care to those deemed less useful in society. As with the other health narratives discussed in this paper, this too mirrors the broader identity narrative of the TPP of a villainous left and victimized public.

At the local level, this narrative circulated at monthly chapter meetings. Activists buzzed with angry excitement, comparing President Obama to Hitler and the ACA to Nazism and pointing to grandma-killing as evidence of these similarities. At protest events Tea Party members were often seen holding signs reading things such as “Obama lies, grandma dies”.

At the national level, the narrative about death panels was perpetuated through online reporting on the Tea Party website and speeches at gatherings. Between 2013 and 2015 twenty-nine articles were posted on the Tea Party website that discussed “death panels”. These included news briefings that mentioned the panels in passing. For example, on February 21, 2015, the Tea Party news brief twice mentioned Obama's mocking of republicans over their belief in death panels. Other examples include articles focusing specifically on death panels, such as an article posted on August 18^th^ called “Democrats Admit Sarah Palin's ‘Death Panels’ are Reality”.

As with the narrative regarding 17^th^-century policies to care for the poor, the death panel narrative is effective because it is unaffected by counter facts. Both Polletta (1998) and Heller (2008) demonstrate the persuasive power of narrative in its ability to withstand alternative reasoning and contradicting evidence [59],[65]. Coercively powerful, the death panel narrative is not falsifiable as it is based on manufactured reality that nonetheless resonates with Tea Party members via their shared culture.

While the death panel, grandma-killing narrative certainly served as an attention gathering and fear provoking rhetorical tool for the Tea Party, it is important to examine its emergence and resonance with members of the party. Specifically, this narrative was developed from a broader cultural logic within the organization. This narrative is rooted in the aforementioned libertarian belief that state intervention necessarily results in a reduction of civil liberties. Moreover, it represents a broader tendency towards, what Hofstadter calls, “paranoia” on the political right wherein adherents employ “heated exaggeration, suspiciousness, and conspiratorial fantasy” to understand and explain social events. As a result of this paranoia, right-wing activists construct extensive and detailed conspiracy narratives that suggest the prevalence of covert entities harming the United States and its citizenship [66]. The Tea Party specifically ties a manufactured narrative about death panels to what they view as the unconstitutionality of the ACA both as a specific bill and it's impact on religious freedom. Activists then pair this legal violation with more egregious crimes such as federally controlled death.

In crafting a story about Constitutionality, Tea Party activists first establish that the Constitution does not give authority to the federal government to mandate things like health care. For example, Tim plainly states: “If we look into Article I, Section 8 of the Constitution it very specifically says to the government—we the people, the bosses—are granting you the authority to do these things and anything you don't see there, you can't do. That is reserve to the states and the people.” Sarah expands Tim's argument: “There is a list of 18 different things on which they [law makers] can legislate. Health care is not there.” Tea Party Activists remain certain that, in Tim's words, “Obamacare will be found completely unconstitutional.” Despite TPP arguments to the contrary, the Supreme Court has upheld the bulk of the provisions of the Affordable Care Act as constitutional. Following this decision, the Tea Party continues to argue that the ACA is unconstitutional. According to the Tea Party Website: “The key features of ‘Obamacare’ [include]… misuse of the Constitution's Commerce Clause.”

Following the logic developed within the constitutionality narrative, Tea Party activists argue that state based programs, such as Mitt Romney's health care program in Massachusetts, provide greater liberty for citizens. According to Tim: “Mitt Romney is living within the constraints of the Constitution. Right, because it was the state… if it doesn't work, it's more easily taken back. Folks don't like what is going on? They can move. Where do we go to, if it is the whole country?” Here Timothy extends his argument interpreting the Constitution to grant extended powers to the states. Importantly, supporting state-based health care still contradicts the logic regarding individual morality outlined by members of the Tea Party. Seemingly aware of this paradox, Tim provides an argument for why state-based health care provides more liberty than a national system: “one can always move.”

The narrative regarding the unconstitutionality of federalized health care and the resultant decrease in civil liberties extends to discussion of Medicare and Medicaid, two distinct programs that are often conflated within Tea Party narratives. Tea Party activists believe that Medicare and Medicaid should be privatized to guarantee their constitutionality and also to increase affordable access. According to Jamie, privatizing these programs will provide increased liberties for consumers: “We say yeah you want to privatize. Yes because you have choices. You won't be forced to not have treatment. You won't be forced to pay a certain amount.” Tea Party activists believe in a central ideology that finds greater transparency and integrity in free market capitalism. In Jamie's words: “Of course we want to go to privatization in things if we can. Will there be some fraud and all that? Yeah. But the market would be all of us here: we're the market. We tell that person don't go to this place they screw you. We don't go so they go out of business. We go to the company that's got the best rates, the best service, the best product, the best choices. That's how it works. That's the free market. Yeah.”

Given that most Tea Party members are older Americans and are either currently qualified or will soon to be eligible for Medicare, this narrative presents an interesting paradox. This paradox may best be exemplified by a sign commonly viewed at Tea Party rallies, which read: “Keep the Federal Government Out of My Medicare”. On the one hand, logical consistency in Tea Party narratives requires activists to seek the privatization of the program, as Jamie argues above. On the other hand, eliminating these programs would hurt Tea Party activists themselves. Thus, within Tea Party narratives, Medicare and Medicaid are framed as entitlement programs, because activists themselves paid into them, and are thus not the same as programs such as the ACA [67]. This, again, highlights the lasting power of cultural narratives in that they can be easily twisted and manipulated to achieve personally beneficial ends.

The Tea Party's logic emerges from this argument as follows: given that the federalization of health care takes power away from people, citizens necessarily lose the ability to make their own decisions regarding health care. From this conclusion, Tea Party activists then interpret the Independent Payment Advisor Panel as a “death panel” making decisions about ones worth to society. According to the Tea Party website, the ACA results in the following:

Decisions about health care, your child's health care, and your elderly parent's health care will now be in the hands of something called the Independent Payment Advisory Board (IPAB). It's a board of fifteen bureaucrats, appointed by the President and unaccountable to you, who will have ultimate authority about all of our health care choices.

Activists, then, extrapolate further, arguing that the death panels provide a slippery slope towards genocide. According to Ann, the ACA infringes on people's religion and morality causing “the morality breakdown”. Ann believes that the ACA will easily expand to state sanctioned killing via death panels: “because every society that has allowed genocide of some sort it transfers to other groups… If you don't value human life which is a natural right then all of a sudden when I turn 80 some panel is going to look at me and say you have our idea of your sense of usefulness is now over how would you like to die?”

Another argument against the Constitutionality of the ACA relies on the political interpretation of the First Amendment. Activists believe that, because the ACA requires contraceptive coverage, it violates the notion of religious freedoms. According to Margaret: “Look at what Obamacare is doing, mandating our religious institutions to provide contraception, sterilization, things of that worth.”

This narrative relies on a libertarian interpretation of the First Amendment. The First Amendment states “Congress shall make no law respecting an establishment of religion, or prohibiting the free exercise thereof; or abridging the freedom of speech, or of the press; or the right of the people peacefully to assemble, and to petition the Government for a redress of grievances” [68]. Neither it nor the Constitution specifically includes the phrase “separation of church and state”. Rather, this phrase was coined in a letter from Thomas Jefferson to the Danbury Baptists, assuring them that the state would not enforce ascription to a particular religion [69]. However, through a series of court cases, the First Amendment has been interpreted to secure a space between the church and the state [70],[71].

Tea Party activists do not agree with the way in which the Amendment has been interpreted in the courts and, instead, believe the Amendment guarantees that the state will not challenge churches. For example, Maxine argues that the First Amendment does not establish the separation of church and state; rather, it establishes religious freedom. Robert, her colleague, suggests that Thomas Jefferson, in his letter to the Danbury Baptists, sought to convince the concerned congregation that the government wouldn't be “interfering with the church” and that it would “protect the church”. Robert suggests that Jefferson and other Founding Fathers were concerned with the government posing a threat to church, not the inverse: “He said the government, if you give them an inch, they'll take a mile. When they take a mile, they're going to weaken the church. They are going to weaken our ability to speak out against those things. They are going to weaken our ability to teach our kids morality.” In this example both Maxine and Robert demonstrate the interpretation of historical facts through a political ideology to advance their narrative.

## 4. Continued Impact of Tea Party Health Narratives

Tea Party health narratives reflect cultural values steeped in libertarian ideology. As a reflection of a cultural worldview, Tea Party health narratives are clearly influencing broader American cultural and political scripts regarding health care and they continue to reflect the cultural beliefs of adherents.

Though not as extensive as Tea Party activists hoped, Tea Party narratives have contributed to the framework of Republican plans to replace the ACA. For example, one such proposal, the American Health Care Act (AHCA), in its initial manifestation by Republicans in the House of Representatives, is deeply rooted in libertarian beliefs [72]. Specifically it seeks to (1) increase the amount individuals or families can place in a Health Savings Account; (2) reduce the Medicaid expansion put in place by the ACA and (3) cut a series of taxes related to the bill (tax penalties for the uninsured, and tax increases on wealthy Americans) [73],[74]. A subsequent draft of the AHCA known as the Better Care Reconciliation Act (BCRA), which has since been rejected by the U.S. Senate, also consisted of several provisions rooted in libertarian ideology such as permitting insurers to sell non-compliant insurance plans outside of the Healthcare Marketplaces and requiring work as a Medicaid eligibility criteria for nonelderly adults [75]. These changes reflect various key areas of Tea Party resistance to the ACA, though, unsurprisingly, some Tea Party activists do not think these bills go far enough to repeal and/or replace the ACA.

Furthermore, Tea Party health narratives reflect continued cultural beliefs of supporters and their allies. Americans have consistently remained divided regarding whether or not the ACA should be repealed and over the specific provisions of the ACA that should be maintained. Among those who continue to call for repeal are Tea Party members whose health narratives continue to resound years after the initial battle over the ACA commenced [76].

Nowhere are similar ideology/partisanship-led belief polarizations more evident than in public opinion polls about healthcare in the U.S. According to polling data, healthcare has remained a major concern for adults in the U.S. In a August 2017 Gallup poll, 17% of the respondents identified healthcare as the “most important problem facing this country today,” ranking just behind “dissatisfaction with the government” (20%), and well ahead of other concerns such as “unemployment” (7%), “immigration” (7%), “economy” (6%), and “situation with North Korea” (4%) [77]. Furthermore, largely as a consequence of the attention given to the repeal of Obamacare throughout and subsequent to the 2016 presidential election, the approval of the ACA has increased: according to Gallup, ACA approval has gone from 42% approving and 53% disapproving in November 2016, to 55% approving and 41% disapproving in April 2017 [78].

This increase in support for the ACA comes after the election of President Trump and reflects a dramatic shift in public opinion. Approval for the ACA remained below 50% throughout Obama's presidency and the corresponding efforts by the Tea Party to fight the legislation. In 2014 only 37% of Americans approved of the ACA. While it is impossible to retroactively determine the degree to which this is a direct result of Tea Party efforts, it is the case that this low approval rating coincides with a two-year period of intensive organizing (in the form of lobbying efforts, online commentary, and public protests) on the part of Tea Party activists against the policy. The Tea Party then became more silent after the provisions went into place until the possibility of repeal reemerged under the Trump Administration.

Notably, the recent increase in approval has come mostly from the “Democratic identifiers and leaners”, who according to Gallup, “have become more hardened in their support for the ACA” [78]. Contrast this with recent data from the Kaiser Family Foundation's Health Tracking Poll [75]. According to this poll, as of May 2017, while 78% of Democrats held *favorable* views about the 2010 healthcare reform bill, a fully 78% of Republicans held *unfavorable* views. These polarized views stand in significant contrast to the views of those who identify as politically independent, among whom, 48% favor the ACA and 45% does not. Partisan and ideological divisions in public attitudes towards healthcare are most prominent in polling questions related to best approaches to handling the ACA. For instance, while 30% of adults generally agree that the ACA should be “repealed and replaced,” the percentage of adults who hold this view is 70% among Republicans and only 4% among Democrats [79]. This corresponds with a drastic drop in support for the Tea Party [80].

## 5. Conclusion

As our case study of the *Tea Party Patriots* demonstrates, Tea Party health narratives are driven mainly by a libertarian ideology that calls into question U.S. healthcare reform (and particularly the ACA) based on several central cultural values: encroachment on individual liberties, values, and rights; creating and increasing government dependence among marginalized and/or undeserving communities; and violating the U.S. Constitution and the First Amendment rights of U.S. citizens. Furthermore, the predominant libertarian ideology and partisanship of the Tea Party health narratives seems to increase polarization within cultural narratives of U.S. healthcare in at least three ways: (1) by intensifying polarization of deeply held values and beliefs, such as views about morality (e.g. ACA mandates as akin to slavery); (2) by generating and disseminating factual misinformation (e.g. conflating facts about Obamacare premiums as a form of government-imposed Tax on the young and the healthy), and (3) by spearheading and disseminating political misperceptions and myths (e.g. Obamacare death panels myth).

The examples presented above provide some justification for one of the leading theoretical explanations for recent trends in political polarization: “party sorting”. It is likely that the ideological healthcare narratives developed by Tea Party activists first exacerbates elite political polarization, as witnessed by the Tea Party influence on Republican healthcare policy. Over time, this elite polarization trickles down to the general public in top-down processes, aided by partisan media outlets (e.g. talk radio, cable TV, think tanks) and Internet platforms such as the Tea Party website, creating deeper divisions in public opinions towards healthcare policy. As the general public faces an increasingly polarized media environment (resulting in echo chambers of like-minded people), Tea Party health narratives provide interpretations that easily fit into and confirm preexisting cultural worldviews, further fueling directionally motivated cognition.

The “party sorting” thesis also sheds light on how certain political misperceptions (i.e. Clinton doctor choice; Obamacare death panels) tend to become sharply polarized over time, aided through the actions of political elites, activists, and media outlets [27]. Accordingly, while the perpetuation of misperceptions is not a problem unique to the political right, the Obamacare “death panel” misperception was observed more prominently among those who identified as Republican. This trend persisted even when controlled for the perceived level of knowledge about ACA. In fact, in Nyhan's work [27], Republicans who believed they had a higher level of knowledge about the ACA were more likely to endorse the “death penal” misperception than those who did not indicate high topical knowledge about the ACA.

Factual misinformation, misperceptions, and ideological narratives such as the Tea Party views discussed above, tend to persist in cultural discourse due to a couple of factors. Firstly, elites and activists on all sides of the political spectrum often succeed in disseminating such views through media. Attempts to correct misinformation become difficult over time, and can even lead to a “backfire effect”. This has prompted some scholars to advocate for (1) stricter media ethics and practices that avoid coverage of serial misinformers; and (2) developing negative publicity for political elites who continue to perpetuate misinformation, in order to combat the spread of political misinformation [27].

Secondly, it is important to recognize that narratives employed by the TPP, such as the “killing grandma” story, do not need a factual basis to be effective in achieving their stated goals (i.e. increasing partisan resistance to the ACA in this case). Instead of aiming to develop a fact-based account of potential ACA failures/weaknesses, the “killing grandma” narrative successfully weaves a believable story about how “government overreach” leads to “death panels” that ultimately “kill society's grandmas.” Such a narrative is persuasive because it provides a well-crafted, affect-generating story that can engage individuals without eliciting a need for fact-based (counter) points [59]. As such, the “killing grandma” narrative accomplishes an “integrative melding of attention, imagery, and feelings” [81], which leads the audience to “focus on the events in the story rather than make counter arguments” [82].

Potential effects of the Tea Party health narratives we have discussed in this article are correlational: we cannot say with certainty how much of a direct influence these narratives have had on public perceptions and/or Republican healthcare policy. However, the persistence of these narratives in the cultural discourse suggests that their potential impacts are worthy of continued scholarly attention.

While we have focused mainly on the likely effects of ideological cultural narratives of the Tea Party on U.S. healthcare policy, future research could extend this line of investigation into other topical areas. It is plausible that Tea Party activism (and other politically motivated social movements on both the political right and the left) continues to shape the larger cultural narrative and public perception around topics ranging from climate change to gun rights. More broadly, future research should examine the impact of cultural narratives on trust in science, trust in the media, and trust in the state.

## Footnotes

1 Nyhan (2010) points to data from a Daily Kos/Research 2000 Poll of 2009 where 11% of Americans believed that President Obama was not born in this country. However, the prevalence of this belief was higher among Republicans (28%). Compare that to a Scripps Howard/Ohio University poll of 2006: while 16% of Americans believed that the Bush administration was complicit in the 9/11 attacks, the prevalence for this belief was 23% among Democrats (Nyhan 2010).
